# Growing the Rhinophorid Tree: Three New Species and Their Phylogenetic Implications (Diptera: Rhinophoridae)

**DOI:** 10.3390/insects11110792

**Published:** 2020-11-12

**Authors:** Silvia Gisondi, Thomas Pape, Hiroshi Shima, Pierfilippo Cerretti

**Affiliations:** 1 Dipartimento di Biologia e Biotecnologie “Charles Darwin”, Sapienza Università di Roma, Piazzale A. Moro 5, 00185 Rome, Italy; silvia.gisondi@uniroma1.it; 2 Natural History Museum of Denmark, Universitetsparken 15, 2100 Copenhagen, Denmark; tpape@snm.ku.dk; 3 Kyushu University Museum, Kyushu University, Hakozaki, Fukuoka 812-8581, Japan; shimarcb@kyudai.jp

**Keywords:** taxonomy, phylogeny, Malaysia, Vietnam, Nigeria, Afrotropical Region, Oriental Region, new species

## Abstract

**Simple Summary:**

Rhinophoridae is a family of oestroid flies which was once considered of strictly Palaearctic distribution. This presupposition has been increasingly contradicted in recent years with the discovery and description of many non-Palaearctic rhinophorid species. In this context, the present research article aims to describe three additional species, namely *Aporeomyia elaphocera* **sp. nov.**, *Baniassa pennata* **sp. nov.** and *Phyto mambilla* **sp. nov.**, from the Oriental and Afrotropical regions. Their genus-level affiliation is supported by a morphology-based phylogeny and these species are described and illustrated, and a comparison with congeners as well as two identification keys are provided. The phylogeny resulting from the addition of these three new species fits well with previous results but retrieves a paraphyletic *Phyto* Robineau-Desvoidy. Notwithstanding this, the authors were taxonomically conservative, avoiding the erection of a new genus for a single species as well as the lumping of three different genera into a single one: the evidence for doing either of such actions is considered premature and more data are needed.

**Abstract:**

Three new species of Rhinophoridae (*Aporeomyia elaphocera* **sp. nov.**, *Baniassa pennata* **sp. nov.** from the Oriental Region, and *Phyto mambilla* **sp. nov.** from the Afrotropical Region) are described, illustrated and compared with congeners. Genus-level affiliation of the new species is based on a morphology-based phylogeny, preliminarily accepting a paraphyletic *Phyto* Robineau-Desvoidy awaiting incorporation of molecular data. Keys to the species of the genus *Aporeomyia* Pape & Shima as well as to the Afrotropical species of the genus *Phyto* Robineau-Desvoidy are given.

## 1. Introduction

Rhinophoridae is a small family of oestroid flies, commonly known as woodlouse flies. Their common name is derived from the trophic habit of their larval stage, as these flies are the only insects that evolved parasitism on non-insect pancrustaceans—i.e., the woodlice (Isopoda: Oniscidea) [1,2,3,4,5,6].

In recent years, research projects concerning rhinophorids have been flourishing, leading to the discovery of several new taxa [7,8,9,10] and the publication of a world catalogue with a key to genera [11]. The present paper aims to describe three new rhinophorid species, which are included in ongoing molecular studies (Gisondi et al.). Their generic affiliation is argued based on morphology-based phylogenetic analyses.

The description of *Aporeomyia elaphocera* **sp. nov.** raises the number of species of the previously monotypic *Aporeomyia* Pape & Shima to two, which allows for a generic definition based on synapomorphies. *Baniassa pennata* **sp. nov.** is the first species of the genus recorded from the Oriental Region, and it is remarkable by a uniquely shaped distiphallus. *Phyto mambilla* **sp. nov.** is the first species of *Phyto* Robineau-Desvoidy from West Africa; it is characterized by a peculiar color pattern and it is only tentatively assigned to *Phyto* Robineau-Desvoidy.

## 2. Materials and Methods

Digital images of the external morphology of the holotypes were taken using a Canon EOS 6D camera equipped with Canon Photo lens MP-E 65mm 1.2.8 and processed by Canon Digital Photo Professional (Canon, Ōta, Tokyo, Japan), Combine ZM by Alan Hadley and GIMP 2.10.4 by Alexandre Prokoudine. Terminology of the external morphology, and measurements and ratios of the head follow Cumming and Wood [12] and Cerretti et al. [11], respectively. We follow Cerretti et al. [11] in using the term “global apomorphy” for an uncontradicted and unreversed apomorphic character state, whereas “local apomorphy” is used for a character state that is homoplasious due to convergence or reversal.

Male terminalia were dissected following the method described by O’Hara [13], and digital images were taken using a DM-LS microscope (Leica, Germany) equipped with a DS-L1 Nikon digital camera (Nikon, Tokyo) and processed using CombineZM (Hadley, UK).

Label data are reported verbatim, with a slash “/” marking the end of each line and a double-slash “//” marking the end of a label. Abbreviations for depositories reported in this work are as follows:

BLKU: Biosystematics Laboratory, Kyushu University, Fukuoka, Japan.
CSCA: California State Collection of Arthropods, Sacramento, CA, USA.
CNC: Canadian National Collection of Insects, Arachnids, and Nematodes, Ottawa, ON, Canada.
MZUR: Museum of Zoology, “Sapienza” Università di Roma, Rome, Italy.
NHMD: Natural History Museum of Denmark, Copenhagen, Denmark.

For the cladistic analysis, the morphological data matrix from Cerretti et al. [11,14] was updated using the software Mesquite [15] to include the three species described here (Matrix S1). Thus, 71 taxa and 100 morphological characters were analyzed through parsimony analyses performed with the TNT software (v1.6-beta) [16]. Characters were treated as unordered and analyzed under implied weighting with a *k*-value ≥ 4 using the “traditional search” algorithm under the following settings: general RAM of 1.0 Gbyte, memory set to hold 1,000,000 trees, 1000 replicates with the tree bisection reconnection (TBR) [17] branch swapping algorithm and saving 1000 trees per replicate. *k*-value was chosen both for consistency reasons with Cerretti et al. [11], who obtained the fittest tree with a *k*-value of 4, but also because low *k*-values (<3) exclude a large amount of information by strongly down weighting homoplasy, whereas larger values (≥3) allow for some signal to come from homoplasy [18]. Branches not discussed in the present paper were collapsed. Consistency and retention indices were calculated using the “States.run” script implemented in TNT.

## 3. Results

### 3.1. Cladistic Results

**Figure 1:**
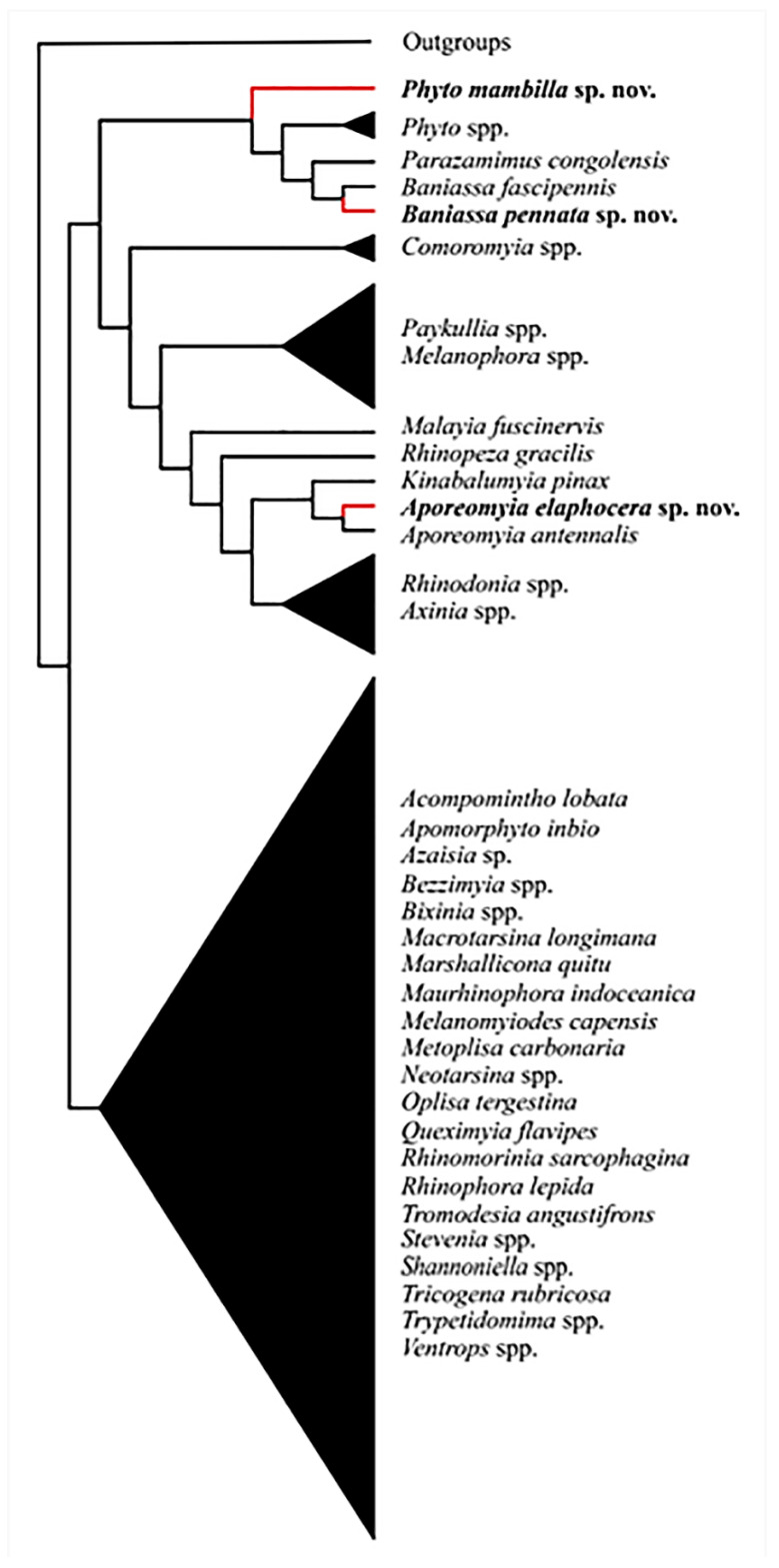
Fittest tree obtained using TNT under implied weighting and a *k*-value ≥ 4.

Analyses yielded one fittest tree (Figure 1). The same topology was retrieved for all *k*-values at or above 4.

*Aporeomyia elaphocera***sp. nov.** clusters with *Aporeomyia antennalis* Pape & Shima based on one global apomorphy (male postpedicel branches from the base into three lobes (Matrix S1: 1:1)) and six local apomorphies (male proclinate orbital setae absent; facial plate deeply sunken; mouthparts strongly reduced; transversal section of sternite 5 almost flat; median extension of surstylus with setae; median process of ventral sclerotization of distiphallus absent (Matrix S1: 10:0; 15:1; 21:1; 55:1; 65:1; 75:1)). *Aporeomyia* Pape & Shima is retrieved as sister to *Kinabalumyia* Cerretti & Pape (see also [11], as *Aporeomyia* sp.), supported by one global apomorphy (first aristomere at least 4× as long as wide (Matrix S1: 7:2)) and two local apomorphies (CuA + CuP reaching wing margin; male tergite 6 fused to syntergosternite 7 + 8 (Matrix S1: 51:1; 59:1)). *Aporeomyia* + *Kinabalumyia* clusters within an Australasian and Oriental clade inside the *Phyto*-group, based on one local apomorphy (arista thickened at least on basal 3/4 (Matrix S1: 6:1)).

*Baniassa pennata***sp. nov.** clusters with *Baniassa fascipennis* Kugler based on four local apomorphies (lappets of metathoracic spiracle unequal in size with the posterior one distinctly larger and closing the spiracle like an operculum; female fore tarsus laterally compressed; setae on median extension of surstyli present; median process of ventral sclerotization of distiphallus not connected to ventral plate (Matrix S1: 31:2; 33:1; 65:1; 76:0)).

*Phyto mambilla***sp. nov.** is recovered within a clade consisting of other species of *Phyto* plus *Parazamimus* and *Baniassa*, and it emerges as sister to the remaining part of this clade—i.e., ((*Phyto adolescens* + *P. angustifrons*) + (*Parazamimus* Verbeke [19] + *Baniassa* Kugler)), based on the lack of setosity in the lower half of the parafacial (Matrix S1: 18:0), thus resulting in a paraphyletic *Phyto*.

### 3.2. Taxonomy

*Aporeomyia* Pape & Shima, 1993.

*Aporeomyia* Pape & Shima, 1993: 77 (original description). Type species: *Aporeomyia antennalis* Pape & Shima, 1993: 77, by original designation.

**References**: [11,20].

**Diagnosis**. Male: Medium-sized, slender flies, with relatively large wing. Head profile narrowed ventrally. Prementum and palpus short. Antenna very large and with postpedicel tripartite from base. Arista inserted on anterior lobe. Prosternum bare. Scutellum slightly swollen. Vein M almost straight and without a bend. Veins M_4_ and CuA + CuP extended to wing margin. Lower calypter narrow and with median margin diverging from scutellum. Hind tibia with posteroventral apical seta.

**Apomorphies**. Male: postpedicel very large and tripartite nearly from base; proclinate orbital setae absent; facial plate deeply sunken; mouthparts strongly reduced; transversal section of sternite 5 almost flat; median extension of surstylus with setae; median process of ventral sclerotization of distiphallus absent.

**Figure 2:**
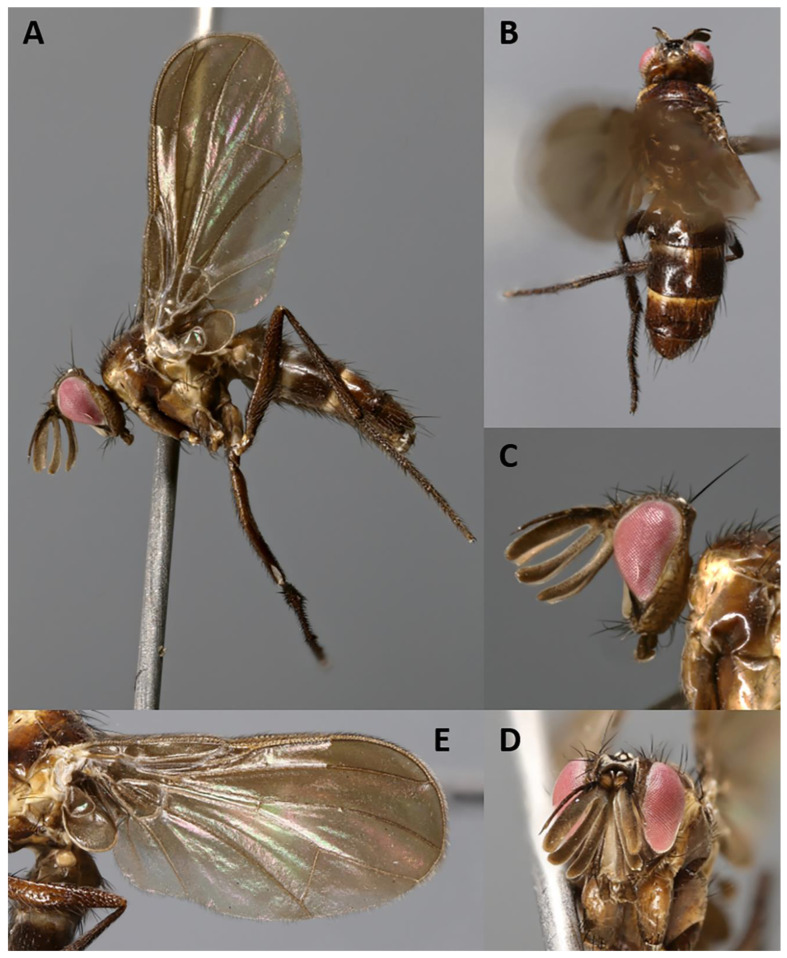
*Aporeomyia elaphocera***sp. nov.** (paratype ♂, MZUR). (**A**) Habitus in lateral view; (**B**) dorsal habitus; (**C**) head in lateral view; (**D**) head in frontal view; (**E**) left wing in ventral view.

**Figure 3:**
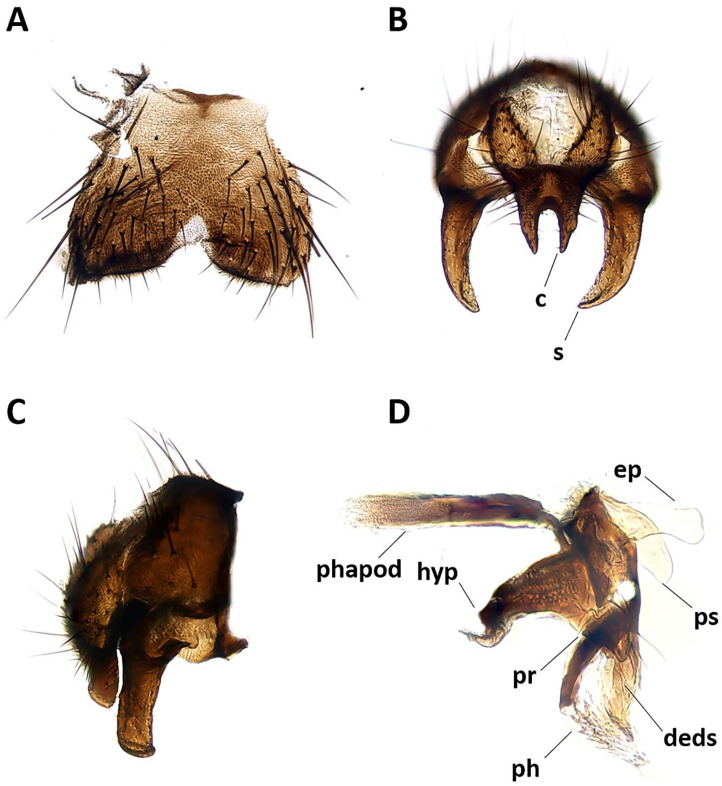
*Aporeomyia elaphocera***sp. nov.**, terminalia (holotype ♂, CSCA). (**A**) Sternite 5 in ventral view; (**B**) epandrial complex in posterior view, c = cercus, s = surstylus; (**C**) epandrial complex in lateral view; (**D**) hypandrial complex and phallus in lateral view, phapod = phallapodeme, deds = dorsal extension of dorsal sclerite of distiphallus, ep = epiphallus, hyp = hypandrium, ph = phallus, pr = pregonite, ps = postgonite.

*Aporeomyia elaphocera* **sp. nov.**

http://www.zoobank.org/A5B08995-E69D-4A5A-B868-AC8655498782 (Figure 2 and Figure 3)

**Type material.** HOLOTYPE ♂: Malaysia, Sabah. Label data: “Malaysia: Sabah: Crocker/Range, Gunung Emas;/5.847000° N 116.322000° E;/26.x-18.xi.2011; S. Bosuang;/Malaise trap; CNC823099.” (CSCA).

**Paratypes:** 3 ♂♂, with label data: “Malaysia, Sabah (Borneo)/Penampang Distr., Crocker Range/Gunung Emas, 1700 m, 15-25. X. 2011/5°49′42″ N 116°20′00″ E, Malaise/M. Hauser & S. Gaimari leg.” (1 CNC, 1 MZUR, 1 NHMD).

**Etymology.** The specific epithet, which should be treated as a noun in apposition, is derived from the specific epithet for the red deer, *Cervus elaphus* Linnaeus, and it refers to the morphology of the postpedicel, tripartite and recalling deer antlers.

**Diagnosis.** Male postpedicel tripartite from base with the three tips running almost straight (not curved as in *A. antennalis* Pape & Shima). Base of vein R_4+5_ bare dorsally. General ground color dark brown (pale brownish in *A. antennalis*).

**Description.** Male (Figure 2 and Figure 3).

Length: 4.5–4.7 mm.

Color (Figure 2). Head mostly brown shading into yellowish towards occipital area; frontal vitta black; area between gena and parafacial blackish; scape and pedicel yellowish to light brown; postpedicel and arista blackish-brown; palpus brown. Scutum and scutellum blackish-brown in ground color; scutum covered with barely visible microtomentum except along three thin pre- and postsutural dark vittae (visible in posterodorsal view); postpronotum yellow; thorax in lateral view with two yellow stripes (anterior one descending from postpronotum to fore coxa, across anterior spiracle, proepisternum and proepimeron; posterior one descending from wing base across anterior half of anepimeron and posterior two-thirds of katepisternum), remaining of pleura brown. Legs dark brown. Upper and lower calypters and wing membrane slightly smoky; tegula brownish and basicosta yellow; wing veins yellow; halter brownish-yellow. Abdomen mostly dark brown in ground color, with anterior portion of tergites 3 and 4 yellowish; tergites 3 and 4 with band of weak, whitish microtomentum on anterior 1/3 (dorsally); terminalia brown.

Head (Figure 2A–D). Head shape receding, roughly triangular in lateral view (i.e., narrowing toward mouthparts). Frons 2.0× as wide as compound eye in dorsal view. Inner vertical seta well developed, 0.8× as long as compound eye height. Outer vertical seta not differentiated. Ocellar triangle with three pairs of short hair-like proclinate setulae (i.e., ocellar seta not differentiated). Frons with 1–4 frontal setae from anterior margin of ocellar triangle and descending at most until middle of pedicel. Fronto-orbital plate with 1 lateroclinate and 3–4 proclinate orbital setae. Upper reclinate orbital setae absent. Parafacial very narrow, as wide as or narrower than width of arista at mid-point. Facial ridge concave with a few fine setulae above vibrissa on lower 1/10 approximately. Vibrissa inserted below level of lower facial margin. Lower facial margin visible in lateral view. Gena approximately 0.3 of compound eye height. Genal dilation weakly developed. Antenna 4× as long as height of gena. Postpedicel tripartite (three branches approximately of equal length), 1.0–1.2× as long as compound eye height. Arista inserted on dorsal branch of postpedicel at 0.15 from base to tip and thickened on nearly whole length. First and second aristomeres elongated, approximately 4–5× as long as wide, both with a seta dorsally. Prementum and palpus very short, almost vestigial.

Thorax. Postpronotum with two setae (Figure 2A). Acrostichal setae absent. One presutural and two postsutural dorsocentral setae. One presutural and one postsutural intra-alar setae. First postsutural supra-alar seta absent. Two + one katepisternal setae. Katepimeron and anepimeron bare. One pair of apical scutellar setae, crossed; one pair of subapical scutellar setae. Anatergite with tuft of short setulae below scutellum. Anterior and posterior fringes of posterior spiracle equal in size and emerging from spiracular rim. *Legs*. Preapical anterodorsal seta of fore tibia about as long as preapical dorsal seta. Mid tibia with one anterodorsal seta. Hind tibia with three preapical dorsal setae. Preapical posteroventral seta of hind tibia about as long as preapical anteroventral seta. Hind tibia with one well-developed anterodorsal seta. Posterodorsal margin of hind coxa bare. *Wing* (Figure 2A,E). Lower calypter tongue-shaped. Second costal section (CS_2_) setulose ventrally. Costal spine not differentiated from other costal setulae. Vein R_1_ bare. Base of vein R_4+5_ bare. Vein M reaching wing margin without bend.

Abdomen (Figure 2A,B). Mid-dorsal depression of syntergite 1 + 2 confined to anterior 1/4 of syntergite. Syntergite 1 + 2 and tergite 3 with two (one per side) median marginal setae. Tergites 4 and 5 each with row of marginal setae. Tergite 5 short, approximately 0.7× as long as tergite 4. Sternite 4 exposed. *Terminalia* (Figure 3). Sternite 5 with narrow V-shaped posteromedian notch without membranous window; anterior margin of sternite 5 about half as wide as posterior margin (Figure 3A). Transversal section of sternite 5 almost flat. Tergite 6 normally developed (i.e., plate-like) with median marginal setae, and fused to syntergosternite 7 + 8. Connection between sternite 6 and syntergosternite 7 + 8 membranous on right side. Epandrium short and convex, with very short anterior prolongation and weakly developed posterior lobe. Cerci normally developed, fused medially at base, distally developed in two pointed, finger-like branches. Surstylus normally developed, relatively narrow and curved inward distally; setae on median extension present. Bacilliform sclerite and surstylus articulated (i.e., not fused). Connection between surstylus and epandrium membranous. Hypandrial arms converging medially, not fused. Connection between phallic guide and pregonite membranous (i.e., not fused). Anterior seta on postgonite absent. Extensions of dorsal sclerite of distiphallus relatively short and partly fused medially to dorsal sclerite of basiphallus. Median process of ventral sclerotization of distiphallus absent. Acrophallus simple, unmodified (apparently with one opening).

**Female.** Unknown

**Distribution.** Oriental: Malaysia (Sabah).

**Biology**. Unknown.

Key to species of *Aporeomyia* Pape & Shima

1. Arista inserted on dorsal branch of postpedicel at about 0.3 from base to tip; first aristomere about as long as wide, second aristomere 2–3× as long as wide. Basicosta dark brown; wing vein R_1_ setulose dorsally on distal 1/2–2/3; vein R_4+5_ setulose dorsally at base and from approximately junction with r-m almost to wing margin. Legs pale brownish yellow.

*Aporeomyia antennalis* Pape & Shima, 1993 (Philippines: Mindanao)

- Arista inserted on dorsal branch of postpedicel at 0.15 from base to tip; both first and second aristomeres elongated, approximately 4–5× as long as wide. Basicosta yellow; wing vein R_1_ bare; vein R_4+5_ bare. Legs dark brown.

*Aporeomyia elaphocera***sp. nov.** (Malaysia: Borneo)

*Baniassa* Kugler, 1978

*Baniassa* Kugler, 1978: 73 (original description). Type species: *Baniassa fascipennis* Kugler, 1978: 74, by original designation.

**References**: [11,14,21,22,23,24].

**Diagnosis**. Male frons narrow, without proclinate orbital and upper reclinate orbital setae. Lunule with setulae. Parafacial varying from nearly bare to almost entirely setulose. Facial ridge bare or with few decumbent setae on lower third. Palpus well developed. Posterior lappet of metathoracic spiracle distinctly larger than anterior lappet. Vein R_1_ bare. Vein R_4+5_ with a few short setulae confined at base or bare. Cell r_4+5_ open or petiolate. Female fore tarsus laterally compressed. Male: Median process of ventral sclerotization of distiphallus interrupted proximally and not connected to ventral plate; surstylus with setae on medial plate.

**Apomorphies**. Posterior lappet of metathoracic spiracle distinctly larger than anterior lappet. Male: Median process of ventral sclerotization of distiphallus interrupted proximally and not connected to ventral plate; surstylus with setae on medial plate. Female: fore tarsus laterally compressed.

**Figure 4:**
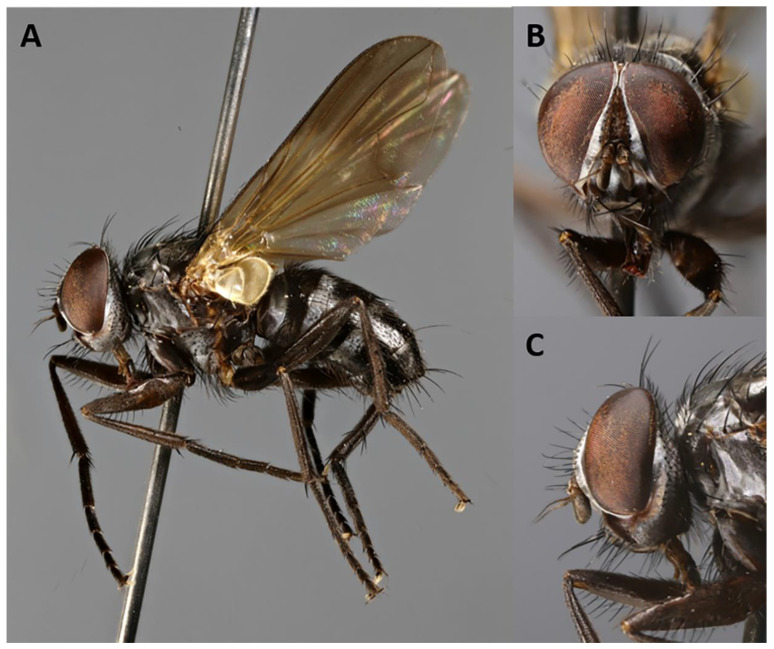
*Baniassa pennata***sp. nov.** (holotype ♂, BLKU). (**A**) Habitus in lateral view; (**B**) head in frontal view; (**C**) head in lateral view.

**Figure 5:**
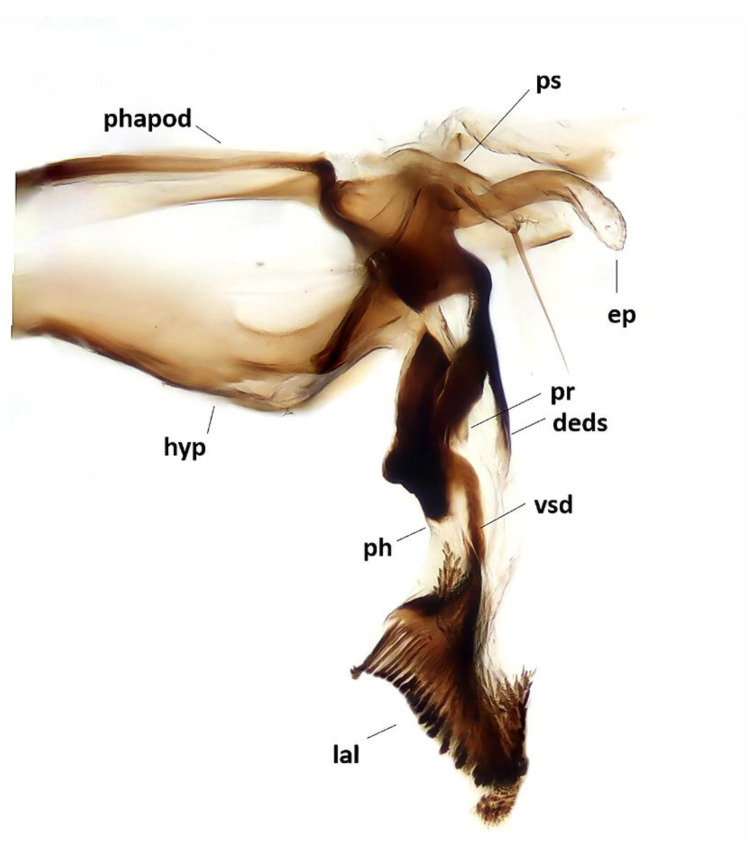
*Baniassa pennata***sp. nov.**, male terminalia (holotype ♂, BLKU): hypandrial complex in lateral view, phapod = phallapodeme, deds = dorsal extension of dorsal sclerite of distiphallus, ep = epiphallus, hyp = hypandrium, lal = lateral lobes, ph = phallus, pr = tip of pregonite, ps = postgonite, vsd = ventral sclerotization of distiphallus.

**Figure 6:**
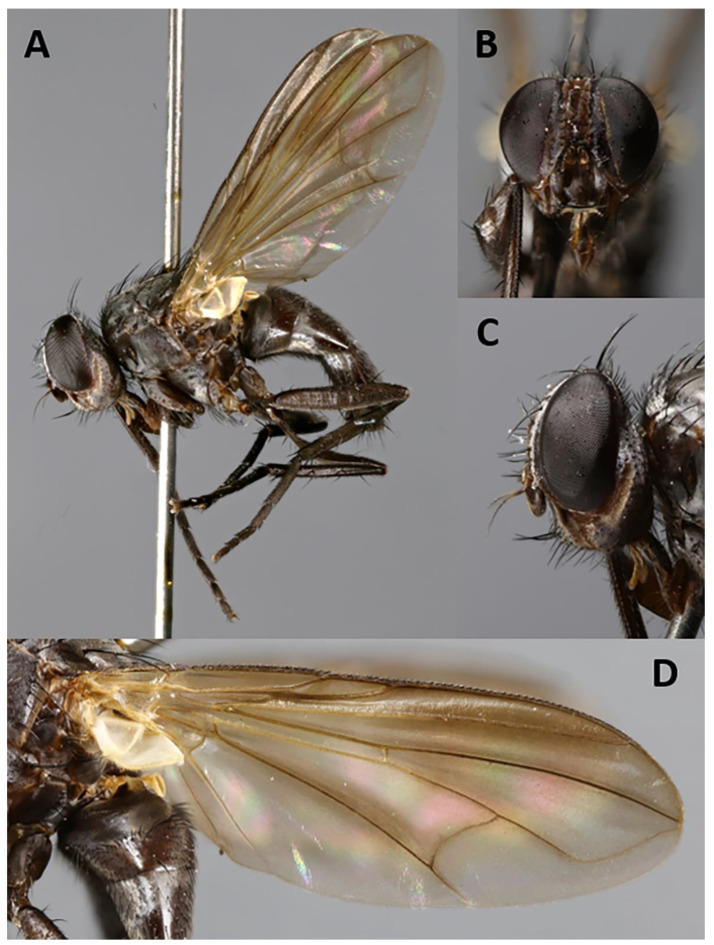
*Baniassa pennata***sp. nov.** (paratype ♀, BLKU). (**A**) Habitus in lateral view; (**B**) head in frontal view; (**C**) head in lateral view; (**D**) left wing in ventral view.

**Figure 7:**
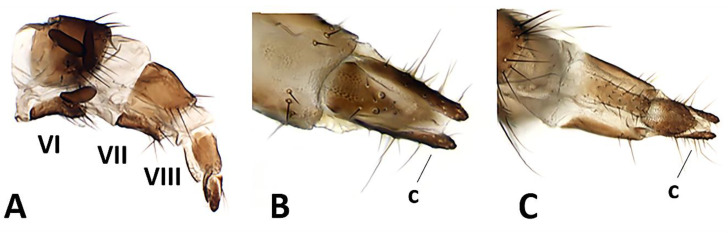
*Baniassa pennata***sp. nov.**, female terminalia (paratype ♀, CSCA). (**A**) Ovipositor in lateral view (showing segments VI, VII, VIII and three spermathecae); (**B**) distal tip of ovipositor in dorsal view, c = cercus; (**C**) distal tip of ovipositor in ventral view, c = cercus.

*Baniassa pennata* **sp. nov.**

http://www.zoobank.org/73D656F3-8F51-4427-871A-850223095F36 (Figure 4, Figure 5, Figure 6 and Figure 7).

**Type material**. HOLOTYPE ♂: Vietnam, Cao Bang Province. Label data: “Vietnam: Cao Bang/Prov., Mt. Pia Oac/1320 m/23–27.v.1999/Col. H. Kurahashi.” (BLKU).

PARATYPES: 3 ♂♂, same data as holotype (1 BLKU, 1 MZUR, 1 NHMD); 2 ♀♀, 1 ♀, same data as holotype (BLKU), 1 ♀, with label data: “Vietnam: Ninh Binh/Prov. Cuc Phuong Natl. /Park, 24–28.iii.2012/20°21′03″ N 105°35′36″ E/SD Gaimari, M Hauser, /HT Pham 390 m.” (CSCA).

**Etymology**. The specific epithet, which should be treated as a Latin adjective, is derived from the Latin “*pennatus*” meaning “feathered”, and it refers to the long, feather-like sclerotizations characterizing the lateral lobes of the distiphallus.

**Diagnosis**. Parafacial bare in ventral half. Postpronotum with three setae. First postsutural supra-alar seta strong, distinctly longer and thicker than notopleural seta. Bend of vein M not reaching vein R_4+5_ so that cell r_4+5_ is open at wing margin. Bacilliform sclerite firmly fused to surstylus. Female thorax ground color black.

**Description**. Male (Figure 4 and Figure 5).

Length: 4.2–4.9 mm.

Head (Figure 4A–C). Frons 1/4 of a compound eye in dorsal view. Inner vertical setae well developed, approximately 0.4× as long as compound eye height. Outer vertical seta not or weakly differentiated from postocular setulae. Ocellar triangle with one pair of proclinate ocellar setae and 3–4 pairs of short hair-like proclinate setulae. Frons with 8–9 frontal setae descending to upper margin of scape. Fronto-orbital plate bare on dorsal 2/3, with minute setulae on distal third, sometimes descending to upper half of parafacial. Upper reclinate orbital setae absent. Proclinate orbital setae absent. Parafacial approximately 0.8–1.1× as wide as postpedicel at mid length, with 1–7 short hair-like setulae on upper half. Facial ridge concave with fine setae above vibrissa at most on lower 1/5. Vibrissa inserted at level of lower facial margin. Face and lower facial margin not visible in lateral view. Gena approximately 0.24–0.27 of compound eye height. Genal dilation well developed. Antenna long, 3/4 of height of gena. Postpedicel 2.0–2.4× as long as pedicel. Arista with minute trichia shorter than its greatest diameter, and thickened on proximal 1/4. First aristomere very short, not longer than wide. Second aristomere slightly larger than first, not longer than wide. Prementum about twice as long as width at mid length. Palpus apically enlarged.

Thorax. Postpronotum with three setae arranged in line of obtuse triangle (Figure 4A). Two presutural and one postsutural acrostichal setae. Two presutural and three postsutural dorsocentral setae. Two postsutural intra-alar setae separated by distance greater than distance between first seta and suture. First postsutural supra-alar seta well developed—i.e., distinctly longer than notopleural setae. Two + one katepisternal setae. Katepimeron with two hair-like setulae. Anepimeral seta short. One pair of apical scutellar setae, crossed. One pair of basal scutellar setae. Anatergite with tuft of short setulae. Anterior and posterior fringes of posterior spiracle unequal in size: posterior lappet distinctly larger. *Legs*. Preapical anterodorsal seta of fore tibia about as long as preapical dorsal seta. Mid tibia with one anterodorsal seta. Hind tibia with three preapical dorsal setae. Preapical posteroventral seta of hind tibia about as long as preapical anteroventral seta. Hind tibia with two well-developed anterodorsal setae. Posterodorsal margin of hind coxa bare. *Wing* (Figure 4A). Second costal section (CS_2_) bare ventrally. Costal spine not differentiated from other costal setulae. Vein R_1_ bare. Base of vein R_4+5_ with 2–4 setulae dorsally and ventrally. Bend of vein M forming obtuse angle. Fourth costal section (CS_4_) longer than sixth (CS_6_). Section of M between crossveins r-m and dm-m 1.1–1.2× longer than section between dm-m and bend of vein M. Cell r_4+5_ open.

Abdomen (Figure 4A). Mid-dorsal depression of syntergite 1 + 2 confined to anterior 1/3 of syntergite. Syntergite 1 + 2 without median marginal setae. Tergite 3 without median marginal and lateral discal setae, with two or three pairs of lateral marginal setae. Tergites 4 and 5 each with row of short marginal setae and without discal setae. Tergite 5 short, approximately 1.3× as long as tergite 4. Sternite 4 exposed. *Terminalia* (Figure 5). Sternite 5 with deep, wide, posteromedian notch with narrow membranous window. Transversal section of sternite 5 U-shaped. Tergite 6 well developed (i.e., plate-like) with median marginal setae. Connection between tergite 6 and syntergosternite 7 + 8 membranous. Connection between sternite 6 and syntergosternite 7 + 8 membranous on right side. Epandrium short and convex; anterior extension well developed, posterolateral lobe scarcely developed. Cerci normally developed, basally wide, not fused medially at base (i.e., suture between cerci complete and visible). Surstylus normally developed, basally wide, narrowing distally (distal third stick-like); setae on median extension present. Bacilliform sclerite and surstylus firmly fused. Connection between surstylus and epandrium membranous. Hypandrial arms converging medially, not fused. Connection between phallic guide and pregonite membranous (i.e., not fused). Anterior seta on postgonite present. Extensions of dorsal sclerite of distiphallus relatively short and partly fused medially to dorsal sclerite of basiphallus. Median process of ventral sclerotization of distiphallus present and not interrupted, running from ventral plate to tip of phallus. Median process of ventral sclerotization of distiphallus longitudinally not divided. Acrophallus simple (i.e., with one opening); lateral lobes of acrophallus with long (feather-like), sclerotized, hooked teeth, resembling a zipper.

**Female** (Figure 6A–D) (4.5 mm). Generally darker in coloration, except wing membrane almost hyaline, differs from males as follows: outer vertical seta present, well developed; one pair of upper reclinate orbital setae; two pairs of proclinate orbital setae; frons approximately 3/4 of a compound eye in dorsal view. *Terminalia* (Figure 7A–C). Oviscapt moderately long and telescopic, retracted in fifth segment (Figure 7A). Segments 7 and 8 normally developed and unmodified. Tergite 8 rectangular and cerci normally developed, straight (Figure 7B,C). Three suboval spermathecae, dark brown in color (Figure 7A).

**Distribution**. Oriental: Vietnam (Cao Bang Province).

**Biology**. Unknown.

*Phyto* Robineau-Desvoidy, 1830

*Phyto* Robineau-Desvoidy, 1830: 218 [original description]. Type species: *Phyto nigra* Robineau-Desvoidy, 1830 (=*Tachina melanocephala* Meigen, 1824), by designation of Townsend (1916:8).

**References**: [25,26,27,28,29,30,31].

**Diagnosis**. Afrotropical species of *Phyto* likely represent a monophyletic group characterized by a distinctive color pattern with contrasting black and silvery grey microtomentose bands (reminiscent of that of the tachinid genus *Trigonospila* Pokorny [32]). Lunule with setae. Parafacial bare. First postsutural supra-alar seta present and well developed, longer than notopleural setae. Male: frons very narrow (narrower than width of postpedicel); fronto-orbital plate without proclinate orbital setae.

**Apomorphies**. None. The present analyses retrieved *Phyto* as a paraphyletic grade from which the ancestor of *Parazamimus* and *Baniassa* arose. The clade composed of *Phyto*, *Parazamimus* and *Baniassa* is supported by a single local apomorphy (lunule with setae). See further under Discussion below.

**Figure 8:**
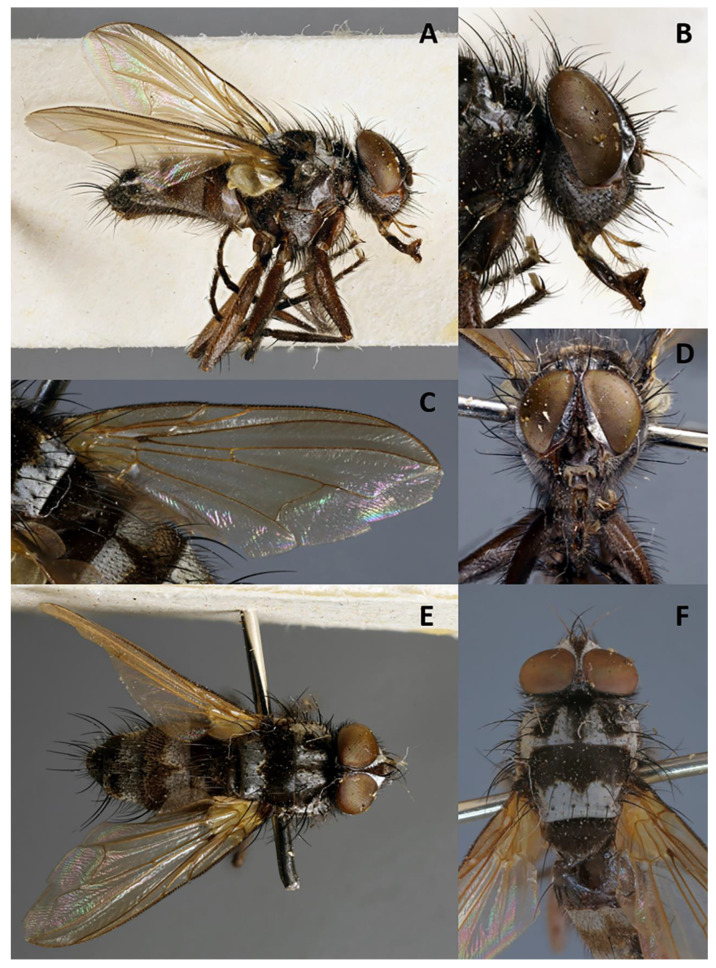
*Phyto mambilla***sp. nov.** (holotype ♂, CNC). (**A**) Habitus in lateral view; (**B**) head in lateral view; (**C**) right wing in dorsal view; (**D**) head in frontal view; (**E**) dorsal habitus; (**F**) thorax in dorsal view.

**Figure 9:**
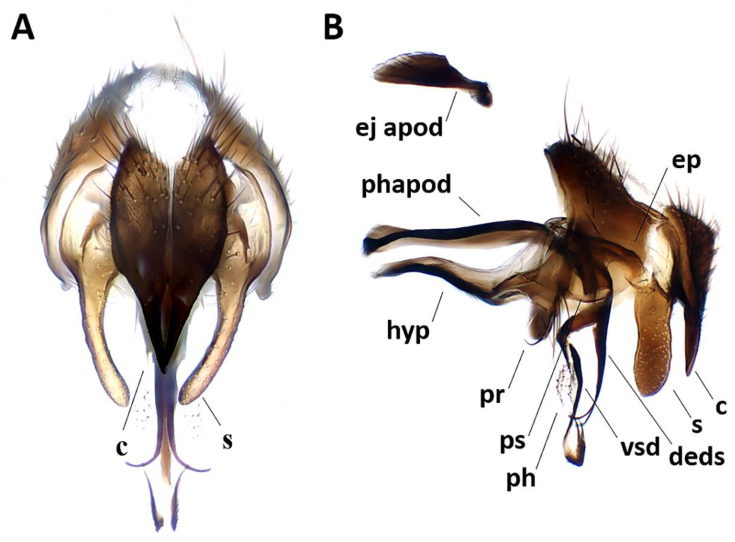
*Phyto mambilla***sp. nov.**, male terminalia (holotype ♂, CNC). (**A**) Cerci and surstyli in posterior view, c = cercus, s = surstylus; (**B**) terminalia, phapod = phallapodeme, c = cercus, deds = dorsal extension of dorsal sclerite of distiphallus, ej apod = ejaculatory apodeme, ep = epiphallus, hyp = hypandrium, ph = phallus, pr = pregonite, ps = postgonite, s = surstylus, vsd = ventral sclerotization of distiphallus.

*Phyto mambilla* **sp. nov.**

http://www.zoobank.org/A1AEBA3F-EBD6-4E8E-BB1C-A6304E9DB56D (Figure 8 and Figure 9)

**Type material**. HOLOTYPE ♂: Nigeria, Gashaka-Gumti National Park. Label data: “Overgrown/cocoyam/plots in/village//Nigeria:/Mambilla/Plateau/Ngel Nyakl./28.xi.-3.xii.1968./J.C. Deeming.” (CNC).

**Etymology**. The specific epithet, which should be treated as a noun in apposition, refers to the type locality.

**Diagnosis.** Male arista with long microtrichia (at most twice the maximum diameter of arista). Parafacial bare in ventral half. Postpronotum with two setae. Bend of vein M not reaching vein R_4+5_ so that cell r_4+5_ is open at wing margin. Katepimeron with few hair-like setulae.

**Description**. Male (Figure 8 and Figure 9).

Length: 4.3 mm.

Color (Figure 8A–F). Head black in ground color and covered with silvery grey microtomentum; frontal vitta brownish; area between gena and parafacial brownish to orange; scape, pedicel and postpedicel dark brown; palpus orangish. Thorax black in ground color; postpronotum silvery grey microtomentose; prescutum with two broad silvery grey microtomentose vittae; anterior 3/5 of scutum black and posterior 2/5 silvery grey microtomentose; scutellum black; anepisternum and katepisternum silvery grey microtomentose, remaining pleura black. Legs light brown shading to dark brown tarsi. Upper and lower calypters whitish. Wing hyaline; tegula and basicosta brown; veins yellow. Halter whitish. Abdomen brown in ground color with two broad silver bands; syntergite 1 + 2 with brown mid-dorsal depression and silver lateral and ventral parts; tergites 3–4 each with silver band on anterior half, almost divided by brown triangle in the middle; terminalia brown.

Head (Figure 8A,B,D–F). Frons at narrowest point much narrower than postpedicel, not more than twice as wide as anterior ocellus, 0.072 of head width and 0.15× as wide as compound eye in dorsal view. Outer vertical seta not differentiated. Ocellar setae well developed, proclinate. Frons with 11 frontal setae descending to upper margin of pedicel. Fronto-orbital plate with few scattered hair-like setulae and frontal vitta obliterated at narrowest point. Upper reclinate orbital setae not differentiated from frontal row. Proclinate orbital setae absent. Parafacial bare, approximately as wide as postpedicel at mid length and well visible in profile. Facial ridge concave with setae above vibrissa on lower 1/5. Vibrissa inserted at level of lower facial margin. Face and lower facial margin not visible in lateral view. Gena approximately 0.3 of compound eye height. Genal dilation well developed. Lunula with setae. Antenna long, 3/4 of height of gena. Postpedicel 2.8–3.0× as long as pedicel. Arista thickened on proximal 1/8, with dorsal and ventral row of fine trichia equally long on upper and lower surfaces, at most 2.5× maximum basal diameter of arista and longest trichia longer than width of frons at narrowest point. First aristomere very short, much shorter than wide; second aristomere not longer than wide. Prementum about twice as long as width at mid length. Palpus apically enlarged.

Thorax. Postpronotum with two setae (Figure 8A,E,F). Two presutural and three postsutural acrostichal setae. Two presutural and three postsutural dorsocentral setae. Three postsutural intra-alar setae. First postsutural supra-alar seta well developed. Two + one katepisternal setae. Katepimeron with few hair-like setulae. Anepimeron with several hair-like setulae. One pair of apical scutellar setae, crossed. One pair of lateral scutellar setae. Anatergite bare. Anterior and posterior fringes of posterior spiracle equal in size, standing out from spiracular rim. Postmetacoxal area membranous. *Legs*. Preapical anterodorsal seta of fore tibia about as long as preapical dorsal seta. Mid tibia with one anterodorsal seta. Hind tibia with three preapical dorsal setae. Preapical posteroventral seta of hind tibia about as long as preapical anteroventral one. Hind tibia with two well-developed anterodorsal setae. Posterodorsal margin of coxa bare. *Wing* (Figure 8C). Second costal section (CS_2_) bare ventrally. Costal spine short but differentiated from other costal setulae. Vein R_1_ bare. Base of vein R_4+5_ with 3–4 setulae dorsally and ventrally. Bend of vein M forming obtuse angle. Fourth costal section (CS_4_) longer than sixth (CS_6_). Section of M between crossveins r-m and dm-m 1.3× longer than section between dm-m and bend of vein M. Cell r_4+5_ closed at wing margin.

Abdomen (Figure 8A,E). Mid-dorsal depression of syntergite 1 + 2 confined to anterior 2/3 of syntergite. Syntergite 1 + 2 without median marginal setae, with one pair of lateral setae. Tergite 3 and 4 with short and recumbent dorsal setulae. Tergite 3 with one pair of median marginal and one pair of median discal setae, and three pairs of lateral marginal setae. Tergites 4 and 5 each with row of marginal setae and without discal setae. Tergite 5 short, approximately 1.4× as long as tergite 4. Sternite 4 exposed. *Terminalia* (Figure 9A,B). Sternite 5 with deep posteromedian notch with sub-basal membranous window. Transversal section of sternite 5 U-shaped. Tergite 6 normally developed (i.e., plate-like) with median marginal setae. Connection between tergite 6 and syntergosternite 7 + 8 membranous. Connection between sternite 6 and syntergosternite 7 + 8 membranous on right side. Epandrium short and convex. Cerci normally developed, not fused medially at base (i.e., suture between cerci complete and visible). Surstylus normally developed, not divided; bifid inner median extension of surstylus absent. Bacilliform sclerite and surstylus articulated and not fused. Connection between surstylus and epandrium membranous. Hypandrial arms converging medially, not fused. Connection between phallic guide and pregonite membranous (i.e., not fused). Anterior seta on postgonite present. Extensions of dorsal sclerite of distiphallus divided into two hemisclerites. Median process of ventral sclerotization of distiphallus present, interrupted proximally and not connected to ventral plate. Median process of ventral sclerotization of distiphallus longitudinally not divided. Acrophallus simple (i.e., with one opening).

**Female.** Unknown.

**Distribution.** Afrotropical: Nigeria.

**Biology.** Unknown.

Key to the Afrotropical species of *Phyto* Robineau-Desvoidy (modified from Crosskey [29])

1. Postsutural area of scutum brownish black. Presutural area of scutum black with two narrow silvery grey microtomentose vittae (vittae laying between rows of acrostichal and dorsocentral setae and separated by black median vitta). Frons at narrowest point 0.080× as wide as head in dorsal view; frontal vitta not obliterated. Parafacial narrow and only just visible on lower half in profile. Two postsutural intra-alar setae, anterior seta closer to suture than to hindmost seta. Two postpronotal setae. Two katepisternal setae.

*Phyto parafacialis* Crosskey, 1977 (South Africa)

- Postsutural area of scutum with following color pattern: posterior third or two-fifths thickly pale yellowish or silver microtomentose (pale microtomentose areas strongly contrasting with black sublateral areas of presutural area and anterior part of postsutural area of scutum). Presutural area either with single wide yellowish microtomentose vitta medially or with two wide subtriangular vittae of silver microtomentum (vittae laying approximately along row of dorsocentral presutural setae); lateral black areas of presutural scutum not reaching transverse suture due to a rim of microtomentum. Frons at narrowest point at most 0.075× as wide as head in dorsal view; frontal vitta obliterated at narrowest point. Parafacial well visible in profile. Usually three postsutural intra-alar setae, median seta (or anterior one if only two present) as close to or closer to hindmost seta than to transverse suture. Two or three postpronotal setae; if three, then arranged in triangle. Three katepisternal setae.

2 (East and West Africa)

2. Presutural portion of scutum with two wide, subtriangular vittae of silver microtomentum (vittae laying approximately along row of dorsocentral presutural setae), separated by black median vitta; lateral black portions of presutural scutum broadly subtriangular in shape and not reaching transverse suture due to a rim of microtomentum. Frons at narrowest point 0.072 of head width.

*Phyto mambilla***sp. nov.** (West Africa)

- Presutural portMedian third of presutural portion and hind margin of scutum thickly pale yellow microtomentose. Frons, at narrowest point, at most 0.065 of head width.

3 (East Africa)

3. Trichia on arista equally long on upper and lower surfaces; longest trichia longer than width of frons at narrowest point. Head ground color entirely blackish, subparafacial area (i.e., portion between genal grove, genal dilation and vibrissal triangle) and frontal vitta area velvety black (not paler than rest of head). Abdominal tergites 3 and 4 with dark color extending forwards as elongate blackish brown triangle on each tergite with apex at anterior margin (separating pale microtomentose basal parts of tergites into two). Dorsal setulae of abdominal tergites 3 and 4 very long, fine and erect. Frons at narrowest point much narrower than postpedicel, not more than twice as wide as anterior ocellus (0.055× head width).

*Phyto paratachinoides* Crosskey, 1977

- Trichia on arista not as long on lower surface as upper surface and longest trichia shorter than width of frons at narrowest point. Head not entirely black; subparafacial area and frontal vitta orange-red, brick-red or reddish brown (i.e., paler than rest of head). Abdominal tergites 3 and 4 with dark areas slightly triangular but not extending forwards in mid line to anterior margins of tergites (bases of tergites therefore with uninterrupted broad pale microtomentose fasciae). Dorsal setulae of abdominal tergites 3 and 4 short and recumbent or virtually so. Frons at narrowest point only slightly narrower than postpedicel, conspicuously more than twice as wide as anterior ocellus (0.065× head width).

*Phyto tachinoides* (Curran, 1927) [33]

## 4. Discussion and Conclusions

*Aporeomyia* is an Oriental genus characterized by a tripartite postpedicel in males. In our analyses, *Aporeomyia elaphocera* **sp. nov.** clusters with *Aporeomyia antennalis* Pape & Shima, and the genus is retrieved as sister to *Kinabalumyia pinax* Cerretti & Pape within an Australasian/Oriental clade. Despite the fact that no female specimens of either species have been found to date, the peculiar antennal morphology likely represents a sexually dimorphic character [20]. Moreover, the discovery of a second species of *Aporeomyia* has given the opportunity to define this genus based on cladistic arguments as detailed above, and the marked difference in postpedicellar morphology between *Aporeomyia* and *Kinabalumyia* is considered here as further support for generic separation.

*Baniassa pennata***sp. nov.** and *Phyto mambilla* **sp. nov.** cluster within a weakly supported Palaearctic/Afrotropical clade.

*Baniassa* is a Saharo-Arabian/Oriental genus, formerly known from only three species restricted in distribution to the Middle East, all of which shared a petiolate wing cell r_4+5_. *Baniassa pennata* **sp. nov.** has the wing cell r_4+5_ open, thus presenting the plesiomorphic condition for this character; moreover, this new species remarkably broadens the genus concept and expands the distributional range to include the Oriental Region [34]. However, we are fully aware that the monophyly of *Baniassa* relies on four weak local apomorphies and that molecular data are needed to better circumscribe the genus.

With 26 described species, the genus *Phyto*, as it is currently defined, is the second most species-rich genus within Rhinophoridae: only *Stevenia* Robineau-Desvoidy comprises more species (28) [11]. *Phyto* mostly shows a west-Palaearctic distribution with only four species in the Afrotropical Region, all endemic: *Phyto mambilla* **sp. nov.**, *P. parafacialis* Crosskey, *P. paratachinoides* Crosskey and *P. tachinoides* (Curran). According to our phylogeny, the phylogenetic position of *Phyto mambilla* **sp. nov.** renders *Phyto* paraphyletic with regard to *Baniassa*. Despite this result, we opted for a conservative classification, avoiding both the erection of a new genus to accommodate *Phyto mambilla* **sp. nov.** as well as the lumping of *Phyto*, *Baniassa* and *Parazamimus* into a single genus, because we consider such action premature, awaiting the imminent results from a comprehensive phylogeny of the Rhinophoridae based on molecular data (Gisondi et al., in prep.). Except for the remarkably different coloration pattern, characterized by white reflecting microtomentum contrasting with the dark integument, the Afrotropical species of *Phyto* are very similar to their Palaearctic congeners. Moreover, the two included Palaearctic species of *Phyto* provide insufficient coverage of the morphological diversity of the genus, and an improved taxon sampling is needed to resolve relationships in this group.

## Supplementary Materials

The following are available online at https://www.mdpi.com/2075-4450/11/11/792/s1, Matrix S1: Morphological data matrix from Cerretti et al. [11,14] modified to include *Aporeomyia elaphocera* **sp. nov.** *Baniassa pennata* **sp. nov.** and *Phyto mambilla* **sp. nov.**
